# Colistin Resistance of* Pseudomonas aeruginosa* Isolated from Snakes in Taiwan

**DOI:** 10.1155/2017/7058396

**Published:** 2017-09-25

**Authors:** Po-Yu Liu, Ling-Ling Weng, Shu-Ying Tseng, Chou-Chen Huang, Ching-Chang Cheng, Yan-Chiao Mao, Kwong-Chung Tung

**Affiliations:** ^1^Department of Internal Medicine, Taichung Veterans General Hospital, Taichung, Taiwan; ^2^Rong Hsing Research Center for Translational Medicine, National Chung Hsing University, Taichung, Taiwan; ^3^Department of Veterinary Medicine, College of Veterinary Medicine, National Chung Hsing University, Taichung, Taiwan; ^4^Veterinary Teaching Hospital, College of Veterinary Medicine, National Chiayi University, Chiayi City, Taiwan; ^5^Laboratory Animal Service Center, Office of Research and Development, China Medical University, Taichung 40402, Taiwan; ^6^Department of Emergency Medicine, Taichung Veterans General Hospital, Taichung 40402, Taiwan

## Abstract

This study included fifty-eight isolates of* P. aeruginosa* from the oral cavity of snakes that were recruited from clinical cases, captive and wild snakes. The minimum inhibitory concentrations (MICs) for the determination of susceptibility were identified by the broth microdilution method. Polymerase chain reaction (PCR) was employed to detect *β*-lactamases genes. With regard to antipseudomonal antibiotics, the lowest nonsusceptible rates were in aztreonam (15%), piperacillin/tazobactam (12%), and amikacin (9%). The nonsusceptible rates were high in gentamicin (33%) and colistin (55%). Meanwhile, *bla*_TEM_ presented in 100% of isolates where *bla*_AmpC_, *bla*_OXA-1_, and *bla*_OXA-10_ came at 94.8%, 89.7%, and 27.6%, respectively. Emergence of multidrug resistant (MDR) strains and colistin-resistant strains highlights the potential breach of public health as* P. aeruginosa* could be transmitted through either direct contact or indirect dissemination through the environment. This study reports that the highly resistant* P. aeruginosa* from snakes' oral cavity were discovered for the very first time in Taiwan.

## 1. Introduction

Antibiotic resistance poses a growing threat to public health all over the globe. Undoubtedly inappropriate prescription is a major issue, and that in agriculture and husbandry deserves more focus. Moreover, surging level of antimicrobial agents employed by veterinary medicine makes animals today potential reservoirs of resistant bacteria [1].

Categorized as opportunistic pathogen, nonetheless,* Pseudomonas aeruginosa* infection could be lethal in the immunocompromised population [2]. Yet the pivotal problem lies in the intrinsic antibiotic resistance of* P. aeruginosa* that counteracts the effects of medical treatments [3]. To current knowledge,* P*.* aeruginosa* is more likely to be an environmental species and passed on by animal reservoirs, the snakes, for instance [3]. In fact, the latest report from Taiwanese Centers of Disease Control illustrates that incidence of snakebites reaches more than 1,000 cases annually [4] and implicates underestimated role of* P. aeruginosa* of snake origin.

However, little is detailed about the determination regarding susceptibility of antibiotics that are widely administered within human population such as piperacillin/tazobactam and meropenem [5]. Since disease-free snakes might be carriers as well, captive ones of commercial origin and from research institution were also enrolled in our study. In brief, sensitivity testing of* P. aeruginosa* from snakes' oral cavities and subsequent molecular typing of resistance genes will be outlined in the following paragraph, with further implication of clinical translation and public health responses.

## 2. Materials and Methods

### 2.1. *Pseudomonas aeruginosa* Strains

The study was implemented based on 58* P*.* aeruginosa* isolates from disease-free captivated snakes, wild snakes at Endemic Species Research Institute (ESRI), and clinical cases at National Chung Hsing University Veterinary Medicine Teaching Hospital, Taichung, Taiwan. All samples were collected through sterilized swabbing and stored in the transport media on the way to microbiology laboratory. Following aerobic culture routine, the isolates were plated onto cetrimide agar and incubated at 35°C in 5% CO2 and 95% air for 48 hours. Based on the acquired colonies,* Pseudomonas aeruginosa* was further determined by colony morphology, Gram stain, oxidase test, and API ID 32 GN strips (bioMérieux, Marcy l'Etoile, France).

### 2.2. Antimicrobial Susceptibility Testing

The minimum inhibitory concentrations (MICs) were identified by Clinical Laboratory Standards Institute (CLSI) [6]. Targeting* P. aeruginosa*, cation-adjusted Müller-Hinton broth was engaged to subsequent serial twofold dilutions of representative antimicrobial agents.* P. aeruginosa *suspension was then adjusted to the turbidity of 0.5 McFarland standard of* P. aeruginosa* ATCC27853, and 100 *μ*L of the bacteria with inoculum density at around 10^5^ CFU/mL was further transferred onto 96-well plates. After incubation at 37°C for 24 hours, MICs of respective antibiotics were then categorized into susceptible, intermediate, and resistant based on the interpretation table within CLSI guidance. The antimicrobials were employed in the study: penicillins (piperacillin/tazobactam), cephems (cefotaxime), aminoglycosides (gentamicin and amikacin), lipopeptides (colistin), monobactams (aztreonam), and carbapenems (meropenem).

### 2.3. Detection of Resistance Genes in* P. aeruginosa* Isolates

The Nucleic Acid Automatic Extraction System (Taco, Taichung, Taiwan) was adopted for DNA isolation through the manufacturer's protocol. The extracts served as PCR templates, where primers and the amplification outcomes of *bla*_TEM_, *bla*_OXA_-group I/III, and *bla*_AmpC_ specific PCR are outlined in Table 1. In brief, 2 *μ*L of DNA template was mixed with 1 *μ*L upstream primer, 1 *μ*L downstream primer, 5 *μ*L of Fast‐Run™ Taq Master Mix (Protech Technology Enterprise Co., Ltd., Taipei, Taiwan), and 16 *μ*L distilled water to reach 25 *μ*L in total. Each premixed set was then anchored within GenePro Thermal Cycler (Bioer Technology, Hangzhou, China). Subsequent to the initial denaturation session of 5 minutes at 95°C, whole PCR was subjected to 30 cycles of 30 seconds at 94°C, half an hour at 55–65°C, 45–75 seconds at 72°C, and final 7-minute-long extension at 72°C.

### 2.4. Statistical Analysis

All data were processed through basic statistic tools of Microsoft Excel (Microsoft Corporation, Redmond, USA).

## 3. Results

### 3.1. Antimicrobial Resistance

MIC distribution of indicative antimicrobial agents is summarized in Table 2, which illustrates further susceptibility translation according to CLSI standards for broth microdilution method and preexisting references of cefotaxime [6]. Of note, only one out of 58 isolates (2%) demonstrated resistance to meropenem otherwise sensitive (MIC ≤ 2 mg/L). Second to meropenem, approximately 80–90% of* P. aeruginosa* isolates also exhibited susceptibility to amikacin, piperacillin/tazobactam, and aztreonam. On the opposite, 52 isolates (90%) were found to be nonsusceptible to cefotaxime, which was followed by colistin at 55% and gentamicin at 33% resistance rate, respectively.

### 3.2. Prevalence of *β*-Lactamase Genes

Meanwhile, *β*-lactamase gene within the context was identified through PCR and subsequent molecular typing as well. Intriguingly,* bla*TEM was discovered among all isolates whereas* bla*AmPC was detected in 55 out of 58 isolates (94.8%). As for Ambler class D *β*-lactamase family, frequency of OXA-1 was over three times higher than that of OXA-10 (89.7% over 27.6%). Remarkably, presence of OXA-10 displayed high correlation (93.8%) with coexistence of other three resistance genes. Distribution pattern of *β*-lactamase genes is abstracted in Table 3.

## 4. Discussion

In our study, antibiotic-resistant* P. aeruginosa* strains from the snakes' oral cavity were identified for the very first time. Of these, resistance rate even reached approximately 50% with certain antibiotics. High prevalence of *β*-lactamases genes was disclosed through our work as well.

In terms of other natural reservoirs,* P. aeruginosa* could only be discovered in a limited number of animals like canine, feline, swan, and reptile [5]. Most studies of animal* P. aeruginosa* strains from Pan-Pacific regions reported relatively subtle resistance [7]. Reportedly, merely 17.8% of* P. aeruginosa* from dogs and cats demonstrated resistance of cefotaxime whereas over half of reptile strains recruited in our study were insensitive to the chemotherapeutic agents [8]. As for carbapenems, it is rather striking to detect 2% resistance in reptile strains as their canine and feline parallels were all sensitive according to current literature [8]. Although polymyxin B resistance was once reported in canine* P. aeruginosa *[9], there exists no direct evidence to implicate colistin (also known as polymyxin E) resistance that was expressed by almost 50% of snake isolates in our study. Combined, to our knowledge,* P. aeruginosa* from snakes' oral cavity are highly antibiotic-resistant.

Indeed* P. aeruginosa* possesses diverse intrinsic resistance mechanisms such as multidrug efflux pumps and antibiotics inactivating enzymes and naturally tolerates a number of antimicrobial agents [10]. Also, the species is likely to develop acquired or adaptive resistance under selective pressure. Although there are certain antipseudomonal antibiotics available, emergence of multidrug resistant (MDR) strains poses a considerable threat to the public health. Since colistin resistance was accidentally discovered through our work, the problem is far more complicated than initially expected.

In Taiwan, MDR* P. aeruginosa* took up 18.6% among all hospital-acquired infections in a retrospective review [11], of which the level was similar to that worldwide. A parallel Taiwanese study also illustrated insufficient colistin susceptibility of* P. aeruginosa,* at a mere 76.7% [12]. Mirrored to our study, colistin resistance of snake* P. aeruginosa* was still 31.7% higher than that of human strains. On the other hand, MDR reptile strains stood at approximately one-tenth in our study, which was similar to 10–20% among human* P. aeruginosa* infections in Taiwan [13].

With regard to antibiotic resistance among* P. aeruginosa* in other Asian-Pacific countries, surveillance report in 2011 outlined the highest carbapenem resistance in Philippines (50%) that was followed by India (32%) and Thailand (30%) [14]. Intriguingly, cross-national colistin susceptibility stood at 98% on average at that time [14].

Applied to clinical aspects, secondary infection of snakebites often arises from mixed pathogen like other animal bites. In a 10-year-long Taiwanese cross-sectional study,* P. aeruginosa* was detected in 23.8% of patients with subsequent infections [15]. In fact, among all Gram-negative aerobes,* P. aeruginosa *was the second most prevalent that followed* M. morganii *and* Enterococcus* species [15].

At the time, there are no guidelines or consensus of antibiotics selection for snakebites [16]. Nonetheless, healthcare professionals should be alerted as MDR and colistin-resistant* P. aeruginosa* strains were recognized in our study. That is, in the context of snakebites in Taiwan, the victim is actually running a high risk of treatment failure.

On the other hand, adaptive colistin resistance of* P. aeruginosa* could also be induced by subinhibitory doses [17]. Surprisingly, the systemic review on pharmacokinetics and pharmacodynamics of colistin is inadequate, even after over-50-year-long clinical adoption [18]. It is foreseeable that colistin administration at a sublethal concentration might be insensible to clinicians and leads to adaptive resistance subsequently. Furthermore, stimulation from colistin at a sublethal level is likely to trigger resistance to aminoglycosides and quinolones [19].

Generated to* P. aeruginosa* of reptile origin, appalling colistin resistance at 55% probably results from exposure to unspecified antibacterial peptides [20]. Concerning serine-*β*-lactamases, 100% prevalence of the resistance gene* bla*TEM among* P. aeruginosa* within natural environments was once reported by Igbinosa et al. before [21], whereas strains extracted from human infection expressed* bla*TEM at a far below ratio [22]. Nonetheless one-tenth of isolates were still susceptible to cefotaxime and the discrepancy between genotypes and phenotypes probably indicated conditional expression of Ambler class A *β*-lactamases genes. In addition, our work highlighted high frequency of* bla*AmpC. Since several antibiotics could upregulate AmpC production [23], none of them were employed in our work in order to avoid batch errors. In other words, route of exposure prior to the recruitment might be the untold story. As all three Ambler classes of *β*-lactamases contributed to cefotaxime resistance, that raised a question about the hierarchy of single resistance gene. In contrast, it could also be possible for a universal cue to orchestrate expression of those genes, simultaneously.

Undoubtedly, certain limitations of our work exist and it takes further studies to discover the missing puzzle pieces. Since meropenem-resistant* P. aeruginosa* was detected, extensive work on genes encoding for carbapenemases such as OXA-48 and metallo-*β*-lactamases is required [24, 25]. Similarly, polymyxin resistance genes, PA4773-PA4775, for example, require thorough investigation [26]. However, impact of resistance genes might be overestimated as they are not constitutive to gene expression. Meanwhile, susceptibility profiles of quinolones should be established in the near future as MDR and colistin resistance among* P. aeruginosa* from snakes' oral cavity was noted.

Of note, oral flora suggests the gut microbiota of the preys, to some extent, as they might defecate even when being consumed [27]. Indeed, any creatures that are exposed to animal performance enhancers (APEs) and in contact with snakes are skeptical culprits. Also, environmental dissemination and unreported preexposures should be taken into account. The authorities should be cautious of disorganized regulation and reporting system of off-label antibiotic administration, of which biochemical characteristics of APEs and other administration details remain unclear as only gross doses in the unit of kilograms and animal species are reported [28, 29]. Otherwise the rooting issue of disseminating antibiotics at a sublethal dose will continue to threaten public health.

## 5. Conclusion

MDR and colistin-resistant* P. aeruginosa* were identified from the snakes' oral cavity in Taiwan for the first time, which indicated high risk of medical treatment failure in the context of snakebites and the crucial role of antibiotic stewardship to prevent adaptive resistance. On the other hand, wide prevalence of serine-*β*-lactamases was also disclosed through our work. Nonetheless the correlation between genotypes and phenotypes still requires further investigation.

## Figures and Tables

**Table 1:** Primers sequences used in the amplification and antimicrobial resistance genes in *P. aeruginosa*.

Class of *β*-lactamases	Primer name	Sequence (5′ to 3′)	Product size (bp)	Target
Class A	TEM-A	GAGTATTCAACATTTCCGTGTC	851	blaTEM
	TEM-B	TAATCAGTGAGGCACCTATCTC		
Class D	OXA-10F	TCTTTCGAGTACGGCATTAGC	760	OXA group I
	OXA-10B	CCAATGATGCCCTCACTTTCC		
	OXA-1A	AGCCGTTAAAATTAAGCCC	911	OXA group III
	OXA-1B	CTTGATTGAAGGGTTGGGCG		
Class C	AmpC-PA1	ATGCAGCCAACGACAAAGG	1243	blaAmpC
	AmpC-PA2	CGCCCTCGCGAGCGCGCTTC		

**Table 2:** Distribution of MICs and antimicrobial sensitivity test of gentamicin, amikacin, cefotaxime, piperacillin/tazobactam, colistin, meropenem, and aztreonam for *P. aeruginosa*.

Agent	Number of isolates with indicated MIC values (mg/L)												Percentage of indicated susceptibility		
	0.125	0.25	0.5	1	2	4	8	16	32	64	128	≥256	*S* (%)	*I* (%)	*R* (%)
Gentamicin	0	0	0	0	5	34	11	2	0	0	1	5	39 (67%)	11 (19%)	8 (14%)
Amikacin	0	0	0	0	0	28	19	6	0	0	0	5	53 (91%)	0	5 (9%)
Cefotaxime	0	0	0	0	0	2	4	18	17	9	6	2	6 (10%)	18 (31%)	34 (59%)
Pip + taz	0	0	0	0	0	14	28	9	5	2	0	0	51 (88%)	7 (12%)	0
Colistin	0	0	0	0	26	25	2	0	2	0	0	3	26 (45%)	25 (43%)	7 (12%)
Meropenem	6	18	8	17	8	1	0	0	0	0	0	0	57 (98%)	1 (2%)	0
Aztreonam	0	0	0	0	4	19	26	6	2	0	0	1	49 (85%)	6 (10%)	3 (5%)

Pip + taz: piperacillin/tazobactam; *S*: susceptible; *I*: intermediate; *R*: resistant.

**Table 3:** Prevalence of Ambler classes A, C, and D *β*-lactamases among fifty-eight clinical isolates of *P. aeruginosa.*

Class	Type of *β*-lactamases	Number (%) of isolates
Class A	blaTEM	58 (100%)
Class D	OXA group I (OXA-1)	52 (89.7%)
	OXA group III (OXA-10)	16 (27.6%)
Class C	blaAmpC	55 (94.8%)
Combined	blaTEM, OXA-1, OXA-10, blaAmpC	15 (25.9%)
	blaTEM, OXA-1, blaAmpC	34 (58.6%)
	blaTEM, blaAmpC	5 (8.6%)
	blaTEM, OXA-10, blaAmpC	1 (1.7%)
	OXA-1, blaAmpC	3 (5.2%)

## Additional Points

*Highlights.* (1)* Pseudomonas aeruginosa* are not uncommon in snakes' oral cavity. (2) Multidrug resistant and colistin-resistant* P. aeruginosa* from snakes' oral cavity were discovered. (3) The lowest resistance rates were in aztreonam, piperacillin/tazobactam, and amikacin. (4) 100% of the isolates harbor resistance gene blaTEM and high frequency of blaAmpC among* P. aeruginosa* isolates.
